# Unpacking teacher collaboration: validation of measurement models using exploratory structural equation modeling

**DOI:** 10.3389/fpsyg.2026.1800096

**Published:** 2026-04-14

**Authors:** Benjamin Waldejer, Maria Therese Jensen, Oddny Judith Solheim

**Affiliations:** 1 The Norwegian Reading Centre, University of Stavanger, Stavanger, Norway; 2 The Norwegian Knowledge Centre for Education, University of Stavanger, Stavanger, Norway

**Keywords:** burnout, exploratory structural equation modelling (ESEM), measurement invariance, teacher, collaboration, work engagement

## Abstract

Teacher collaboration represents a key organizational feature of primary education, yet empirical research in the Nordic context has been limited by a lack of validated instruments capable of distinguishing between different forms of collaborative practice. This study develops and evaluates a set of measures designed to capture multiple dimensions of teacher collaboration in Norwegian primary schools. Scale development was informed by qualitative interviews with teachers from four schools spanning lower and upper primary grades. The instrument was administered to 490 teachers at Time 1 and 380 teachers at Time 2 across 38 schools. Exploratory structural equation modeling (ESEM) was used to examine the dimensionality and coherence of the proposed measurement models. Results supported two multidimensional factor structures. Construct validity was supported by evidence of criterion-related validity, examined through associations with burnout, engagement, and school contextual variables. Longitudinal analyses assessed reliability, temporal stability, and measurement invariance across teacher subgroups and measurement occasions using current procedures for ordinal data. The findings indicate that the proposed measures demonstrate satisfactory psychometric properties and stable factor structures over time. The study provides researchers and educational practitioners with robust tools for the systematic investigation of teacher collaboration, its organizational conditions, and professional outcomes in primary education.

## 1 Introduction

Teacher collaboration matters. Teacher collaboration is widely recognized as a critical component of school improvement and organizational development (Kolleck et al., 2021; Muckenthaler et al., 2020). Empirical evidence suggests that collaborative practices among teachers contribute positively to both student achievement and teacher well-being (Mora-Ruano et al., 2018; Reeves et al., 2017), while also mitigating professional isolation and fostering the exchange of pedagogical knowledge (Tallman, 2021). Although international research on teacher collaboration has increased in recent years, significant gaps remain regarding the specific conditions that enable sustained and meaningful collaborations. Moreover, the multidimensional nature of collaboration presents challenges for its conceptualization and measurement. This study addresses these issues by introducing and validating a novel instrument designed to assess teacher collaboration within the Nordic educational context.

Historically, teaching has been marked by professional isolation, with collaboration viewed as an exception rather than the norm (Lortie, 1975). This individualistic model, often described as the “egg-crate” system, reflected a compartmentalized school structure where classroom practices were opaque and disconnected (Lortie, 1975; Tyack, 1974). While such autonomy may benefit some highly skilled educators, it limits opportunities for shared learning and systemic coherence. As educational demands grow increasingly complex, collaboration among teachers has shifted from a discretionary practice to a structural necessity. It is now widely recognized as a key factor in promoting transparency, professional growth, and overall school quality (Azorín and Fullan, 2022; Hargreaves and O’Connor, 2018; Robinson, 2011). Despite this shift, our understanding of what enables effective collaboration across diverse school contexts remains incomplete.

Recent review studies have emphasized the link between school culture, shared teacher values, and educational quality. In his synthesis of 30 years of research, Hargreaves (2019), highlights contrived collegiality as one of three central issues towards developing high-quality schools. This concept underscores the cultural shifts needed to make collaboration the norm rather than the exception. In a comprehensive review, Vangrieken et al. (2015) introduced team entitativity as a framework for understanding varying degrees of collaboration within teacher teams – from loosely connected groups to fully integrated structures. Their findings also point to individualistic school cultures, marked by autonomy and independence, as major barriers to deeper collaboration. Similarly, García-Martínez et al. (2021) conclude that the success of collaborative practices depends on leadership strategies, interpersonal relationships, and shared professional values.

Few validated instruments currently exist for studying teacher collaboration. However, recent developments, particularly within the German educational context, have advanced both its conceptualization and measurement (Hartmann et al., 2020; Richter and Pant, 2016; Schuster et al., 2021). A widely adopted typology of collaboration, grounded in subjective psychological theory and earlier conceptual frameworks (Little, 1990), was introduced by Gräsel et al. (2006) and has since become a reference point in Central European research (Drossel et al., 2019; Krammer et al., 2018; Maag Merki et al., 2022). Building on this theoretical foundation, Fussangel (2008) developed a typological scale to assess the prevalence of different forms of collaboration, which has been applied in subsequent empirical studies (Muckenthaler et al., 2020).

Teacher well-being has become a central concern in educational research, and collaboration is increasingly recognized as a factor that may influence teachers’ emotional and motivational experiences. Burnout is an essential aspect of teacher well-being and can be defined as, a state of emotional, mental, and physical exhaustion caused by prolonged stress, where demands consistently exceed a person’s resources, in turn leading to reduced well-being, motivation, and functioning. Evidence from other professional sectors, such as healthcare, suggests that collaborative practices can reduce burnout by distributing responsibilities, fostering support, and enhancing professional functioning (Kanayama et al., 2016; Ostovar-Nameghi and Sheikhahmadi, 2016). In educational contexts, some forms of collaboration may also increase stress, particularly when interactions are perceived as obligatory, involve strained interpersonal relationships, or are poorly aligned with professional goals (Hargreaves, 2019; Meredith et al., 2020). Together, these findings point to a potentially complex relationship between collaboration and burnout that remains insufficiently explored in schools. Similarly, research on the links between teacher collaboration and engagement – a positive state of well-being characterized by high energy, involvement, and a sense of meaning – remains limited, despite theoretical claims that supportive professional interactions can foster motivation, commitment, and professional efficacy.

Previous research has suggested important links between school contextual variables – such as collegial support and shared goals and values – and teachers’ willingness and motivation to collaborate, which in turn may contribute to improved instructional quality (Skaalvik and Skaalvik, 2021; Vangrieken et al., 2015). While positive collegial relations and social support are well-established contextual correlates of teacher collaboration, less is known about the role of supervisory support, despite frequent suggestions in the leadership literature that leadership is linked to collaborative teacher cultures (Kolleck, 2019; Weddle, 2022). However, it remains unclear whether supervisory support is distinct from, or adds uniquely to, supervisors’ active leadership in facilitating teacher collaboration. Moreover, time pressure has been shown to be related to supervisory support (Skaalvik and Skaalvik, 2009; Vangrieken et al., 2015) and therefore represents an additional relevant contextual factor. Taken together, these school contextual variables and their associations with teacher collaboration form an important nomological network that warrants empirical examination.

As Hargreaves (2019) notes, the field lacks a comprehensive inventory for analyzing collaborations systematically. Also, national education systems shape how teacher collaboration unfolds, making the adaptation of existing measures to local contexts a necessary step (Liu and Benoliel, 2022). In the Scandinavian context, quantitative research on teacher collaboration remains scarce, and no validated instrument currently distinguishes between types of collaboration in a way that reflects institutional conditions. To address this gap, the present study develops and validates a measure based on the typological framework (Gräsel et al., 2006), tailored to the Nordic educational context.

### 1.1 Defining collaboration in terms of measurement

Teacher collaboration has been conceptualized in various ways over the past decades. While a detailed analysis of definitions is beyond the scope of this study, a brief overview of key perspectives is necessary to operationalize the concept. In a systematic review of terminology, Vangrieken et al. (2015) describe teacher collaboration as an “umbrella term” encompassing joint interactions required to perform shared tasks, noting that collaboration can vary in depth. This broad definition serves as a general reference point. For analytical purposes, however, this study also adopts a narrower definition, focusing on observable collaborative behaviors and acts, to facilitate a more precise study of collaboration types.

One of the most influential frameworks for understanding teacher collaboration comes from Little (1990), who categorized collaborative behaviors along a continuum from individual independence to collegial interdependence. Four key forms were identified: storytelling and scanning, aid and assistance, sharing, and joint work. The first, storytelling and scanning, refers to informal and sporadic exchanges of teaching experiences, maintaining full professional autonomy. Aid and assistance involve collegial support but with individual independence. Sharing denotes more structured exchanges, often tied to organizational development. At the most interdependent end of the continuum is joint work, where teachers engage in shared tasks with collective responsibility.

A more common definition of collaborations found in recent European studies on teacher collaborations, is the typology of teacher collaborations, which was conceptualized by Gräsel et al. (2006). Based on subjective psychological theory, collaborations are defined in terms of varying degrees of *cost*, in other words, perceived negative consequences of engaging in tasks (Eccles and Wigfield, 2002). Exchange refers to the day-to-day practical exchanges of information or materials among colleagues needed for teaching, synchronization refers to the need for coordinating joint tasks and division of labor, while co-construction refers to the joint creation of curriculum or collective learning activities. The two former are considered low cost collaborations that do not require any forms of negotiated positions, while the latter is considered high cost, involving professional decision making and deeper learning activities (Gräsel et al., 2006).

Three distinct operationalizations of the typology proposed by Gräsel et al. (2006) have been applied in studies using German-language samples. Fussangel (2008) developed a comprehensive set of scales that differentiate types of collaboration and specify whether they occur across schools or within subject-specific groups. The instrument also includes related dimensions such as organizational structures and perceived benefits. Keller-Schneider and Stefan (2013) introduced scales that capture specific cooperative acts, including the frequency of co-teaching. More recently, Grosche et al. (2020) refined the concept of co-construction, which was further developed and validated as a measurement tool by Kluge et al. (2025). Several other instruments have been developed to assess teacher collaboration in various educational contexts. The Teacher Collegiality Scale (Shah, 2011) is a multidimensional tool consisting of seven components, validated in schools in Pakistan. The TALIS surveys have included a brief measure with two dimensions: collaboration related to teaching and practical coordination (Carlsten et al., 2020). In Norway, a six-item scale has been used to assess perceived benefits of collaboration, though it does not distinguish between types of collaboration (Lerang et al., 2021; Mudhar et al., 2024). Another instrument used in Norway is a four-item scale developed by Munthe (2003), based on theoretical work by Little (1982) outlining key features of professional collaboration.

Although several instruments exist to assess collaboration and related constructs, important limitations warrant the development of a new measure. Contextual differences across national educational institutions limit the transferability of existing scales and affect which dimensions are substantively relevant. Moreover, restricted response formats (e.g., four-category scales) constrain variability and can impair latent variable estimation. The existing instrument based on Gräsel et al.’s (2006) typology (Fussangel, 2008; Muckenthaler et al., 2020) assesses whether practices “apply” rather than their frequency, thereby limiting the conceptualization of collaboration in terms of behavioral acts. Finally, existing measures are often too lengthy or inflexible for inclusion in larger surveys, underscoring the need for brief, psychometrically sound scales.

### 1.2 The present study

The present study develops and validates two multidimensional scales measuring perceived aspects of teacher collaborations in the Nordic primary education context, adapting existing typologies to capture multiple facets of collaborative practice. The study addresses three research questions:

RQ1. To what extent do the proposed measurement models of teacher collaboration demonstrate coherent and temporally stabile factor structures?

RQ2. Do the latent constructs exhibit convergent and discriminant validity when examined in relation to external variables and theoretically relevant outcomes?

RQ3. Does the measurement model demonstrate threshold, weak factorial, and strong factorial invariance across teacher subgroups and measurement occasions and are the measurements reliable?

### 1.3 Validity framework and research question rationale

Modern validity theory treats validity as a unified concept rather than separate types, emphasizing construct validity as cumulative evidence supporting a measure’s intended interpretation (Cronbach, 1980; Messick, 1989). Validity depends on the population, context, and purpose of measurement and incorporates multiple sources of evidence, including content, criterion, convergent, and discriminant relationships, as well as consequences of test use. Following these principles, the present scales are developed from prior conceptualizations of teacher collaboration, literature review, and interviews with experienced educators to ensure contextual relevance. Item generation integrates existing instruments with insights from practice, and scale development follows Netemeyer et al. (2003) framework, which emphasizes cumulative evidence for overall validity throughout construction. Quantitative validation focuses on the dimensional structure of teacher collaboration and its associations with teacher outcomes. Teacher collaboration is modeled as a latent construct underlying observed item responses, with careful scale design to minimize measurement error and common validity threats such as fatigue, misinterpretation, or social desirability.

To evaluate the structure and meaning of the scales, we use Exploratory Structural Equation Modeling (ESEM), which accommodates multidimensional constructs while providing confirmatory model-based estimates. Convergent and discriminant validity are examined through associations with theoretically relevant constructs, including organizational and interpersonal variables and teacher outcomes. Ordinal measurement and measurement invariance across teacher subgroups and timepoints are assessed using procedures appropriate for categorical data (Liu et al., 2017; Wu and Estabrook, 2016), ensuring that observed differences reflect substantive variations in collaboration rather than artifacts of response scaling.

Against this theoretical background, the research questions are justified as follows. RQ1 addresses the need for an empirically supported representation of the construct’s dimensionality. Collaboration practices span multiple, interrelated dimensions, making it important to identify whether proposed dimensions are statistically distinguishable while acknowledging that items may relate to more than one facet of the construct. ESEM provides an appropriate framework because it integrates the flexibility of exploratory factor analysis with the confirmatory capabilities of structural equation modeling, allowing the dimensional structure to be tested while retaining model-based fit indices and parameter estimates. Establishing a well-supported ESEM model provides foundational evidence of construct validity and ensures that subsequent analyses are grounded in a measurement structure that reflects the complexity of collaborative practices.

RQ2 focuses on the meaning of the resulting latent factors and their theoretically expected relationships with other constructs. Convergent validity is supported when dimensions of teacher collaboration are associated with conceptually related professional or instructional variables, whereas discriminant validity is indicated when weak relations emerge with constructs that theory suggests should be unrelated. The ESEM framework ensures that these associations are examined using factor scores that reflect distinct dimensions rather than artificially isolated or overly correlated representations. Embedding the scales within a nomological network, we examine relationships with organizational factors (e.g., time pressure, supervisory support, shared goals) and interpersonal resources (e.g., collegial support), as well as relevant criteria such as burnout and engagement, thereby clarifying the substantive meaning of the dimensions and providing critical evidence that the measure captures targeted aspects of teacher collaboration.

RQ3 concerns the comparability of scores across groups and time. Because the instrument is based on ordinal response formats, demonstrating measurement invariance requires methods appropriate for categorical indicators. Differences in threshold parameters across groups or time can otherwise be misinterpreted as differences in the latent construct rather than shifts in response styles or item functioning. Following Wu and Estabrook’s (2016) and Liu et al.’s (2017) recommendations, we assess invariance by comparing models that sequentially constrain factor loadings and thresholds, rather than intercepts, to ensure that the latent structure operates equivalently across teacher subgroups and measurement occasions. Establishing this form of invariance is essential for defensible comparisons of teacher collaboration, as it can confirm if observed differences reflect substantive variations in collaborative practices.

## 2 Methods

### 2.1 Transparency statement

This study is part of the Teaching Together project, pre-registered on the Open Science Framework (OSF | Teaching Together). A designated data administrator anonymized the dataset before transfer, ensuring transparency and compliance with Norwegian data protection regulations; ID keys and the raw dataset will be kept until the end of the project, and then deleted. The dataset used here, with certain background variables (e.g., school ID) removed, will be made available in the OSF repository alongside a codebook and R scripts for full replicability. Some negatively framed items were reverse coded prior to analyses, and missing values or duplicates in background variables were removed. Sparse or empty categories were collapsed in line with Distefano et al. (2021) to enable estimation of invariance models across groups and for one of the longitudinal ESEMs^1^. The project was reviewed by Sikt (ref. nos. 682183 and 866552) and REK Vest (ref. no. 717284), which concluded that the study did not require review under Norwegian health research legislation.

### 2.2 Samples and participants

This study followed a mixed-methods approach, consistent with recommendations for scale development. Initial item development was informed by a literature review, interviews with teachers from the target population, and consultations with a panel of school leaders and administrators to ensure content relevance and face validity. Feedback from the panel was incorporated into a draft questionnaire, which was piloted prior to distribution to the main sample.

For the main survey, teachers were recruited from 38 schools within a large Norwegian municipality. At T1, 491 teachers participated (mean age = 41, SD = 11.34; 75% female), and 389 participated at T2 (mean age = 39.53, SD = 10.33; 72% female), with 164 teachers completing both waves. Average teaching experience was 12.08 years (SD = 9.74) at T1 and 11.32 years (SD = 9.59) at T2. The mean grade level taught was 5.6 at T1 (SD = 3.2) and 4.8 at T2 (SD = 2.64). Teachers on short term contracts (less than 2 weeks) or various forms of leave were excluded from the sample. Participation was voluntary, fully anonymized, and approved through informed consent.

### 2.3 Questionnaire development

To ensure contextual relevance and face validity, item generation began with consultations involving school leaders, administrators, and experienced teachers from the target municipality. These consultations informed two continua for school selection: (1) pedagogical orientation, from progressive to traditional, and (2) the extent of systematic efforts to develop professional learning communities. Two schools from each primary education level (lower, middle, upper) were selected, and two to three teachers per school were recruited, considering age and gender balance, though the sample was predominantly female.

Semi-structured, open-ended interviews (Brinkmann and Kvale, 2015, 2018) guided by literature-derived topics were conducted, transcribed, and inductively coded in NVivo. Themes were refined based on theoretical relevance and empirical support. Constructs identified for the questionnaire included collaborative structures, culture of sharing, collaborative leadership, types of collaboration, collaborative climate, mutual dependence, team goals and values, perceived efficiency, and contrived collegiality.

Item development followed Podsakoff et al.’s (2003) guidelines to minimize method bias. Items were designed to capture individual experiences, attitudes, and perceptions; phrasing was kept short, clear, and unambiguous, avoiding double-barreled or leading questions. Negatively framed items and neutral instructions were included to balance response tendencies. Items were grouped by construct to reduce cognitive load, maintain conceptual clarity, and minimize fatigue, particularly given teachers’ time constraints. Collaboration items were drawn from interview data, while outcome and validation measures were sourced from existing instruments to reduce common-method bias.

The initial item pool for teacher collaboration included 64 items. The final questionnaire contained 168 items, including background, grouping, outcome, and auxiliary variables, with all but background and grouping items using Likert-type scales. A pilot study tested survey platform functionality (Nettskjema, UiO) and data administration procedures.

### 2.4 Teacher collaboration construct domains

The questionnaire was designed to capture multiple facets of teacher collaboration, organized around nine content domains. All items were scaled on 5-point Likert-type scales ranging from “Very rarely” to “Very often” or “Completely disagree” to “Completely agree.” Collaborative structures encompass the formal and informal organizational arrangements that support joint planning and teaching, including scheduled team meetings, cross-grade coordination, and defined roles for collaborative work. A sample item is “In my school we have scheduled time for teacher collaboration at the grade level”.

Culture of sharing reflects norms and practices that facilitate the exchange of pedagogical knowledge and teaching materials, fostering openness and trust in professional interactions. A sample item is “At our school, we care about sharing with each other.” Collaborative leadership addresses how school leaders enable and sustain collaboration, including providing guidance, resources, and modeling effective collaborative practices. A sample item is “The management is committed to facilitating good collaboration processes.”

Types of Collaboration (Gräsel et al., 2006) differentiates interactions by intensity and perceived “cost,” (Eccles and Wigfield, 2002) ranging from routine exchanges of information (exchange), coordinated joint tasks and division of labor (synchronization), to deeper joint work on curriculum, shared problem-solving and collective learning (co-construction). Sample items are “In my team we meet to coordinate who does what” for exchange, “In my team we meet to schedule when different tasks are going to be done” for synchronization and “In my team we together reflect about lessons” for co-construction.

Collaborative climate captures the broader interpersonal and organizational environment, including trust, psychological safety, and collective valuation of collaborative practice. A sample item is “I feel secure, and I do not fear making mistakes when I collaborate with others on my team.” Mutual dependence reflects teachers’ perceptions of interdependence, shared responsibility, and reliance on colleagues’ expertise. A sample item is “I am not able to do good work without collaborating with my team.” Team goals and values assesses alignment in educational priorities, pedagogical approaches, and professional values among staff. A sample item is “In my team we work in the same way regarding assessments and grading.” Perceived efficiency measures teachers’ evaluation of the utility and productivity of collaboration, considering whether it enhances effectiveness without undue burden. A sample item is “In my team we get a lot done or produced during the time we collaborate.” Finally, contrived collegiality captures instances of collaboration that are formally mandated but lack genuine engagement, reflecting participation driven by obligation rather than intrinsic motivation. A sample item is “I feel forced to work in a team even though I do not want to.” Together, these domains provide a comprehensive framework for operationalizing and measuring teacher collaboration in the Nordic primary education context.

### 2.5 External measures to assess evidence of validity

To assess criterion validity, we included the 12-item Burnout Assessment Tool, BAT (Schaufeli and De Witte, 2023) and the 9-item Utrecht Work Engagement Scale, UWES, short form (Schaufeli et al., 2006) in the questionnaire. Emotional Exhaustion (5 items) from Maslach Burnout Inventory, MBI (Maslach et al., 1997) was also added, as previous research indicates it is particularly sensitive to social support (Jensen and Solheim, 2020, p. 373), making it relevant for examining teacher collaboration. BAT was developed to address limitations of MBI and provides a multidimensional assessment of burnout. It includes exhaustion, capturing the experience of extreme physical and emotional fatigue due to work demands; mental distance, reflecting a sense of detachment or disengagement from work responsibilities; cognitive impairment, which refers to difficulties with concentration, memory, or problem-solving linked to stress; and emotional impairment, indicating a reduced capacity to manage or regulate emotional responses in work situations.

UWES short form measures work engagement independently of burnout (Schaufeli et al., 2006), avoiding conceptual overlap and allowing engagement to be conceptualized as a positive, distinct construct. It includes vigor, reflecting high levels of energy and resilience while working; dedication, capturing a sense of significance, pride, and enthusiasm in one’s work; and absorption, describing being fully concentrated and happily engrossed in work tasks. BAT items use a 5-point Likert-type scale, whereas UWES items use a 7-point Likert-type scale. Together, these measures provide a balanced assessment of teachers’ experiences of both work-related strain and positive engagement, enabling analyses of how different dimensions of teacher collaboration relate to well-being and motivation.

To evaluate further evidence of concurrent and discriminant validity related to external variables, a set of established scales were included in the study. These measures collectively contribute to constructing a nomological network surrounding teacher collaboration. A 5-item scale for supervisory support (Skaalvik and Skaalvik, 2009) was included to discriminate between leader support and collaborative leadership. Additional constructs, shared goals and values (Skaalvik and Skaalvik, 2021) and collegial support (Skaalvik and Skaalvik, 2011) both of which measured with 3 items each, were selected to assess concurrent and discriminant validity in relation to collaborative culture. To further strengthen the network a 5-item scale of time pressure (Skaalvik and Skaalvik, 2011) was included. Together, these constructs provide a theoretically grounded framework for evaluating the validity of the collaboration measure within a broader system of related work and social dynamics.

### 2.6 Analytical approach

Data analyses and modelling were conducted in R, primarily using the packages psych (Revelle and Revelle, 2015) and lavaan (Rosseel, 2012)^2^. Given the ordinal nature of the variables, initial data exploration focused on univariate skewness, kurtosis, and category distributions to guide estimation, convergence, and scale evaluation. Descriptive statistics from psych() indicated non-normality, and further testing with the “discnorm” package confirmed the absence of multivariate latent normality (Cain et al., 2017; Foldnes and Grønneberg, 2020a; Foldnes and Grønneberg, 2020b; Foldnes and Grønneberg, 2022; Micceri, 1989). Outliers were not excluded, as responses were assumed genuine considering that the respondents were all adult professionals in the field of education and there was no reason to doubt the authenticity of responses (Hair et al., 2019).

Factor structures were explored via polychoric correlations, parallel analysis, and exploratory graph analysis, with oblique rotation due to expected factor intercorrelations (Brown, 2015; Morin et al., 2013). Items were iteratively removed for cross-loadings (≥0.30), insufficient loading differences (<0.20), or low communalities (<0.40), maintaining factor stability (Costello and Osborne, 2005; DeVellis, 2017; Field, 2013; Hair et al., 2019; Henson and Roberts, 2006; Netemeyer et al., 2003; Tabachnick and Fidell, 2020).

Exploratory structural equation modeling (ESEM) was employed to allow cross-loadings and provide robust model fit indices (Asparouhov et al., 2018; Asparouhov and Muthén, 2009; Silvestrin, 2020). Set-ESEM was applied to theoretically related item groups to reduce parameter inflation (Marsh and Alamer, 2024). WLSMV was the primary estimator, with DWLS and bootstrapping as secondary options (Brown, 2015; Hancock and Mueller, 2013; Kline, 2023). Theta parameterization and polychoric correlations accounted for ordinal data (Muthén and Muthén, 1998–2017). ESEM measurement models were also compared to CFA models for scale development guidance.

Model fit was evaluated using relative *χ*^2^ (*χ*^2^/df < 2–3), CFI, TLI, RMSEA, and SRMR, following established guidelines with some flexibility for ordinal data (Byrne, 2013; Hu and Bentler, 1999; Xia and Yang, 2019). Modification indices (>10) were applied only when theoretically justified. All code for ESEM and prediction modeling will be made available in the project repository at OSF following publication.

Measurement invariance was tested across sex and age groups (<35, 35–50, >50) using ΔCFI (≤ 0.01), ΔRMSEA (≤ 0.015), and scaled chi-square difference tests (Rutkowski and Svetina, 2014; Wu and Estabrook, 2016). ICCs at the school level were examined to evaluate group-level measurement properties. Longitudinal invariance was tested using threshold and loading constraints (Liu et al., 2017). Reliability was assessed via inter-item and item-total correlations, with McDonald’s omega as the primary indicator (Kalkbrenner, 2023; Marsh et al., 2014; Orçan, 2023; Stensen and Lydersen, 2022).

Longitudinal missing data were handled with pairwise-present estimation, maximizing data use without imputing values, acknowledging that unbiased estimates require MCAR (Rosseel, 2012b). Model modification was conservative to avoid overfitting, with residual covariances between factors and items across time points permitted in longitudinal models (Little, 2024).

## 3 Results

### 3.1 RQ1. Factor structures and dimensionality

As traditional tests of factorability, such as the Kaiser-Meyer-Olkin index and Bartlett’s test of sphericity, are not suitable for ordinal data, we therefore assessed factorability by inspecting polychoric correlation matrices. The clustered correlation plots revealed clear and theoretically coherent patterns, with items forming distinct groups consistent with the proposed typology of collaboration. These clusters also showed expected associations with related constructs, providing preliminary support for the hypothesized measurement structure. Based on these patterns, we selected 45 items that demonstrated the strongest convergence with the proposed constructs: 24 items targeting the three collaboration types described by Gräsel et al. (2006) – exchange, synchronization, and co-construction—six items assessing contrived collegiality, nine reflecting collaborative school culture, and six capturing collaborative leadership. Some redundancy among the collaboration-type items was anticipated, given that only three underlying dimensions were hypothesized.

Dimensionality reduction guided item removal based on cross-loadings, low loadings, and insufficient communalities, with cross-loadings largely attributable to overly similar item phrasing. To avoid artificial clustering, exploratory graph analysis (EGA) and factor analysis were conducted separately for two item sets: the first capturing types of collaboration and the second assessing related constructs.

The 24 collaboration-type items initially produced divergent solutions – five factors from the scree plot, seven from parallel analysis, and six from EGA – while the 21 items in the second set suggested four factors (with parallel analysis indicating five). Items were iteratively removed according to prespecified criteria; however, theoretically important items were retained when they contributed to factor interpretability and stability. To ensure meaningful latent structures, a minimum of three items per factor was required for retention.

Repeated extraction yielded a stable three-factor solution for collaboration types – exchange, synchronization, and co-construction – and a corresponding three-factor solution for the second item set, comprising collaborative leadership, collaborative culture, and contrived collegiality. Both final models demonstrated clear factor structures and accounted for a satisfactory proportion of variance. Based on results from the exploratory factor analyses, the final item pool consisted of 10 items for the collaboration types model and 15 items for the second set of related constructs.

### 3.2 Exploratory structural equation models

#### 3.2.1 Set 1: types of teacher collaboration

Following item reduction via EFA, we estimated a three-factor structure aligned with the theorized dimensions using ESEM. The use of ESEM mitigates the conceptual challenge of exploring and confirming measurement structures within the same sample, while also permitting the modeling of relations with outcome variables. This analytic strategy enabled a comprehensive examination of convergent and discriminant validity within the newly developed scales and relative to established external measures. It further allowed a more detailed investigation of cross-construct item relations.

Due to differences in estimation between the packages psych and lavaan, the patterns of loadings had some minor differences, leading to certain adjustments based on findings with the ESEM model. First, attempts at modelling showed an excellent fit with most indices. However, RMSEA was above desirable levels, indicating misfit. On closer inspection, one of the variables related to synchronization had a loading that was excessively high (0.97). We therefore exchanged it with one of the items that proved redundant in the EFA. This item also showed excessive loading (0.92). However, the new model had an acceptable RMSEA, and all other indices showed an excellent fit to the data. Still, the model showed cross-loadings slightly above the established cutoffs for one item. However, this item was retained for purposes of factor stability and due to theoretical importance.

Responses were left-skewed with few responses in the lowest end of the scale, indicating a high degree of collaborative behavior among respondents in the sample. However, there were sufficient responses for model convergence, model fit was excellent with 67 parameters and 18 degrees of freedom (χ^2^/df 3.22, robust CFI = 0.979, robust TLI = 0.948, robust RMSEA = 0.076, SRMR = 0.024). Standardized covariances of the factors are relatively high (0.65, 0.67, 0.69), indicating that there may be some redundancy between factors. Inter-set correlations showed clear relations between the variables (*r* ≈ 0.10–0.50), however none of them were strong enough to indicate multicollinearity. The result indicates that there may be a common multidimensional factor explaining the shared variance. ICCs using school as a clustering variable were mostly below 0.1, although two items were above at 0.15 and 0.11, which shows that there is less variance between clusters than within.

**Figure 1:**
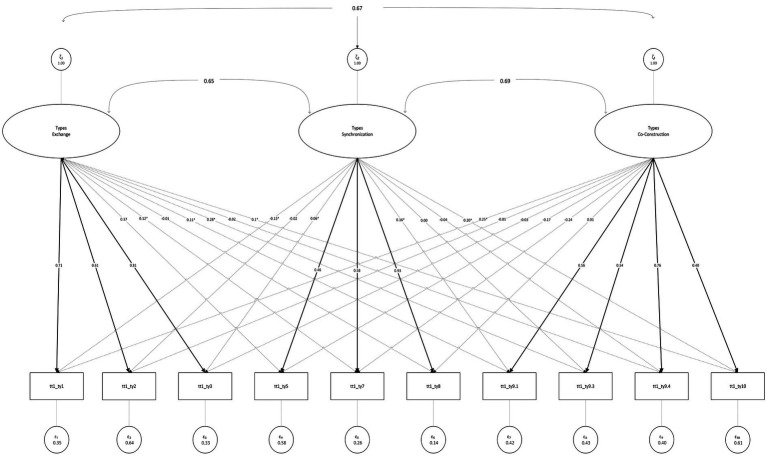
ESEM factor diagram of collaboration types.

A longitudinal model with all three collaboration constructs, using two waves, showed an acceptable fit with 149 parameters and 120 degrees of freedom (*χ*^2^/df 1.59, robust CFI = 0.944, robust TLI = 0.911, robust RMSEA = 0.084, SRMR = 0.048). Longitudinal autoregressions were significant for all three constructs, with high standardized betas indicating a high degree of longitudinal stability. Exchange showed a standardized autoregressive coefficient of 0.746, synchronization 0.781, and co-construction demonstrated particularly high temporal stability with a coefficient of 0.866. Primary factor loadings were mostly strong in CFA but differed a bit in comparison to the ESEM. As expected, loadings were somewhat lower in ESEM due to the estimation of cross-loadings, but remained within acceptable ranges, supporting the construct validity of the factors (see Table 1; Figure 1).

**Table 1:** Collaboration types factor loadings ESEM and CFA comparison.

Constructs	Items	ESEM Std. λ	ESEM $h^{2}$	CFA Std. λ	CFA $h^{2}$
Exchange	In my team we share information that is necessary for teaching	0.71	0.50	0.81	0.66
	I get important messages on time	0.61	0.37	0.59	0.35
	I get learning materials from other teachers in my team	0.81	0.65	0.81	0.66
Synchronization	In my team we delegate tasks that later are done individually	0.47	0.22	0.62	0.38
	In my team we meet to schedule when different tasks are going to be done	0.58	0.34	0.85	0.72
	In my team we meet to coordinate our plans and tasks	0.93	0.86	0.90	0.81
Co-construction	In my team we together create common goals	0.56	0.31	0.77	0.59
	In my team we together create new lesson plans	0.54	0.29	0.76	0.58
	In my team we together create common routines	0.76	0.58	0.72	0.52
	In my team we reflect together about lessons	0.40	0.16	0.64	0.41

ESEM model fit (n.obs = 491, estimator = WLSMV): χ^2^/df 3.22, robust CFI = 0.979, robust TLI = 0.948, robust RMSEA = 0.076, SRMR = 0.024.

CFA model fit (n.obs = 491, estimator = WLSMV): χ^2^/df 3.28, robust CFI = 0.960, robust TLI = 0.944, robust RMSEA = 0.079, SRMR = 0.035.

#### 3.2.2 Set 2: related constructs

As with the first item set, a three-factor structure was pursued based on the theorized constructs. Further item reduction due to model misfit resulted in a final ESEM model comprising 12 items, with each factor represented by four indicators. Six items were developed to measure collaborative leadership, defined as the extent to which school leaders facilitate and support teacher collaboration, including involvement in academic processes and time allocation. Four items were retained in the final model, establishing collaborative leadership as a distinct factor. Nine items were developed to measure collaborative culture, which was operationalized as shared goals and values related to collaborations. Four items were retained, forming a distinct factor. Six items were designed to assess contrived collegiality, defined as teachers’ perceptions of time use, benefit, and comfort in collaborative settings. Four items were retained in the final model. The final model demonstrated good fit across all indices (χ^2^/df = 3.163, robust CFI = 0.984, robust TLI = 0.967, robust RMSEA = 0.062, SRMR = 0.022), supporting the adequacy of the three-factor structure. For both contrived collegiality and collaborative culture, all items had ICCs below 0.1. However, for collaborative leadership, two of the four items were above at 0.17 and 0.12.

Longitudinal modelling of the three factors across two waves initially showed non-convergence. Inspection of modification indices and the residual matrix indicated that one of the four items belonging to the collaborative culture construct exhibited strong cross-wave residual correlations. After removing this item and re-estimating the model using DWLS with mean- and variance-adjusted scaling, the model converged and demonstrated very good fit (164 free parameters; scaled χ^2^/df = 1.71, scaled CFI = 0.993, scaled TLI = 0.993, scaled RMSEA = 0.032, SRMR = 0.046).

**Figure 2:**
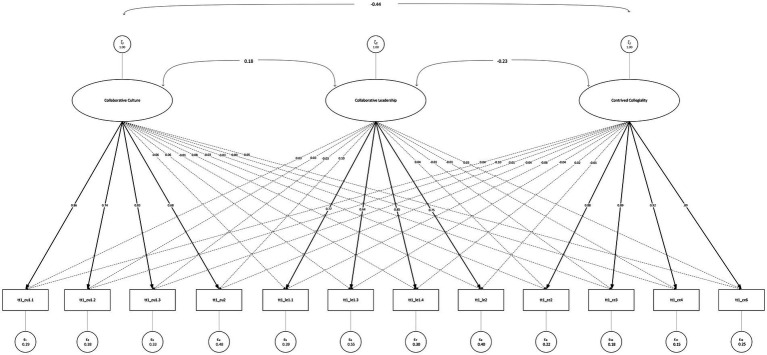
ESEM factor diagram of related constructs.

All autoregressive paths were statistically significant, although the level of longitudinal stability differed substantially across constructs. Contrived collegiality showed high temporal stability (*β* = 0.82), suggesting a relatively trait-like pattern. In contrast, collaborative leadership (*β* = 0.35) and collaborative culture (*β* = 0.43) showed more modest stability, indicating that these constructs may fluctuate more in response to situational or contextual changes over time. Because the model could not be estimated with the WLSMV estimator^3^, robust standard errors were not available, resulting in larger SEs and therefore increased uncertainty around parameter estimates (see Table 2; Figure 2).

**Table 2:** Related constructs factor loadings ESEM and CFA comparison.

Constructs	Items	ESEM Std. λ	ESEM $h^{2}$	CFA Std. λ	CFA $h^{2}$
Collaborative culture	In my team we work on the same topics simultaneously	0.86	0.74	0.83	0.68
	In my team we use the same teaching methods	0.74	0.55	0.81	0.66
	In my team we work in the same way regarding assessments and grading	0.83	0.69	0.80	0.64
	The subject teachers at the grade level work toward the same academic goals	0.68	0.46	0.74	0.54
Collaborative leadership	The school leaders are important in supporting team collaborations	0.77	0.59	0.77	0.59
	The school leaders are involved in academic collaborations	0.64	0.41	0.69	0.47
	The school leaders are involved in collaboration regarding classes	0.85	0.72	0.81	0.66
	The school leaders are committed to facilitating well-functioning collaboration processes	0.75	0.56	0.79	0.62
Contrived collegiality	I do not benefit from working in a team	0.88	0.77	0.88	0.78
	It is a burden for me to have to work in a team	0.89	0.79	0.91	0.82
	To work in a team is a waste of my time	0.92	0.85	0.92	0.85
	I feel forced to work in a team even though I do not want to	0.89	0.79	0.86	0.74

ESEM model fit (n.obs = 491, estimator = WLSMV): χ^2^/df 3.16, robust CFI = 0.984, robust TLI = 0.967, robust RMSEA = 0.062, SRMR = 0.022.

CFA model fit (n.obs = 491, estimator = WLSMV): χ^2^/df 2.17, robust CFI = 0.977, robust TLI = 0.970, robust RMSEA = 0.059, SRMR = 0.040.

### 3.3 RQ2. Criterion-related validity

#### 3.3.1 Set ESEM 1 with outcomes

All outcome measures (BAT, UWES, Emotional exhaustion) underwent CFA before being included in ESEM models as outcomes. BAT showed excellent fit with a freely correlated first-order model (*χ*^2^/df = 1.96, CFI = 0.998, TLI = 0.997, RMSEA = 0.044, SRMR = 0.044) with strong loadings (>0.6). UWES9 showed convergence issues, particularly for the Absorption subscale, consistent with prior observations of prevalent covariances (Seppälä et al., 2009). Modification indices were used to add covariances until acceptable fit, but full-scale ESEM models still faced convergence problems. Therefore, the ultrashort UWES3 version was used, showing acceptable fit (χ^2^/df = 4.88, Robust CFI = 0.997, Robust TLI = 0.973, Robust RMSEA = 0.092, SRMR = 0.022). Emotional exhaustion required a covariance between its first two items to achieve acceptable fit (χ^2^/df = 1.94, CFI = 1.0, TLI = 0.999, RMSEA = 0.044, SRMR = 0.017), likely reflecting similar item wording.

Auxiliary constructs underwent standard CFA. Time pressure showed acceptable fit despite some misfit: χ^2^/df = 7.44, robust CFI = 0.954, robust TLI = 0.909, robust RMSEA = 0.131, SRMR = 0.039. Shared goals and values could not converge as a standalone CFA due to few items and high inter-item correlations but performed well within the full ESEM (loadings >0.70). Collegial support exhibited a Heywood-type solution due to very high loadings (>0.90), yet the factor was stable in the full model. Supervisory support showed strong loadings (0.80–0.86); standalone CFA fit was poor (robust RMSEA ≈ 0.16), but performance improved in the full ESEM, indicating the misfit was estimation-related rather than substantive.

To assess evidence of concurrent validity, a type of criterion-related validity, collaboration types (set 1) were modelled with BAT as outcome variables. Both independent and dependent measurement models were freely correlated. Due to convergence issues, regression paths for each dimension of BAT had to be modelled separately. Modelling collaboration types with the first dimension of BAT, exhaustion, model fit was acceptable: χ^2^/df = 2.486, robust CFI = 0.962, robust TLI = 0.934, robust RMSEA = 0.077, SRMR = 0.034. Exchange had the clearest relation to exhaustion with a standardized regression coefficient of −0.461 at the < 0.01 significance level. Synchronization had a modest coefficient of 0.269 at the < 0.05 significance level. Co-construction had no significant relation to Exhaustion.

Modelling collaboration types with the second dimension of BAT, mental distancing, model fit was acceptable: χ^2^/df = 2.239, robust CFI = 0.969, robust TLI = 0.946, robust RMSEA = 0.067, SRMR = 0.034. A similar pattern of standardized regression coefficients was found. Exchange had a moderate negative coefficient of −0.402 at the < 0.01 significance level, while synchronization had a moderate positive coefficient of 0.336 at the < 0.05 significance level. Co-construction showed no significant path.

Modelling collaboration types with cognitive impairment, showed that model fit was good, but no significant regression paths were observed. For emotional impairment, all paths were significant at the < 0.05 level and the model showed good fit: χ^2^/df = 2.274, robust CFI = 0.973, robust TLI = 0.954, robust RMSEA = 0.065, SRMR = 0.035. Exchange had a small negative coefficient of −0.259, synchronization had a moderate positive coefficient of 0.313, and co-construction had a small negative coefficient of −0.254.

For emotional exhaustion from MBI with collaboration types, model fit was acceptable but with a slightly elevated RMSEA: χ^2^/df = 3.76, robust CFI = 0.943, robust TLI = 0.915, robust RMSEA = 0.087, SRMR = 0.037. Exchange showed a moderate negative coefficient of −0.317 at the < 0.01 significance level, while synchronization had a moderate positive coefficient of 0.305 at the < 0.05 significance level. Co-construction did not show a significant path.

For the model with UWES3 and collaboration types, fit was excellent: χ^2^/df = 1.696, robust CFI = 0.982, robust TLI = 0.968, robust RMSEA = 0.05, SRMR = 0.030. However, only one regression path was significant, co-construction showed a small positive standardized regression coefficient of 0.238 at the < 0.05 significance level.

#### 3.3.2 Set 2 ESEM with outcomes

Modelling the second set-ESEM with BAT as outcome, the model showed a good fit to the data: χ^2^/df = 1.922, robust CFI = 0.964, robust TLI = 0.953, robust RMSEA = 0.055, SRMR = 0.038. For exhaustion, both contrived collegiality and collaborative leadership showed significant associations, with standardized regression coefficients of former with a positive direction 0.186 (*p* < 0.01) and the latter in negative direction with −0.193 (*p* < 0.001). Collaborative culture did not show a significant relationship. Mental distancing was significantly positively related to contrived collegiality (*β* = 0.345, *p* < 0.001) and negatively associated with collaborative leadership (*β* = −0.247, *p* < 0.001), while collaborative culture again showed no significant relationship. Cognitive impairment was significantly positively associated with contrived collegiality (*β* = 0.233, *p* < 0.001) and negatively with collaborative leadership (*β* = −0.139, *p* < 0.01). No significant relationship was found with collaborative culture. Emotional impairment was also significantly positively associated with contrived collegiality (*β* = 0.204, *p* < 0.01) and negatively with collaborative leadership (*β* = −0.162, *p* < 0.01), with collaborative culture showing no significant path.

The model with emotional exhaustion from MBI had an acceptable model fit: χ^2^/df = 3.204, robust CFI = 0.950, robust TLI = 0.929, robust RMSEA = 0.080, SRMR = 0.038. Emotional exhaustion (MBI) was significantly related to two of the constructs. Contrived collegiality had a positive relation to emotional exhaustion (*β* = 0.257, *p* < 0.001). Collaborative culture showed a significant negative relationship, with an estimate of −0.144 (*β* = −0.137, *p* = 0.004). Collaborative leadership did not have a significant relation to emotional exhaustion (*β* = 0.038, *p* = 0.491).

For the model with UWES3, the model fit the data well: χ^2^/df = 1.714, robust CFI = 0.984, robust TLI = 0.974, robust RMSEA = 0.046, SRMR = 0.028. Work engagement (UWES3) was significantly related to two constructs. Contrived collegiality had a negative relationship to work engagement (*β* = −0.194, *p* = 0.001), suggesting that higher levels of contrived collegiality are associated with lower engagement. Collaborative culture also showed a significant positive relationship (*β* = 0.190, *p* < 0.001), indicating that a stronger collaborative culture is linked to higher work engagement. Collaborative leadership did not have a significant relationship with work engagement (*β* = 0.079, *p* = 0.165).

The collaborative leadership scale was compared with supervisory support, an established measure of leader support in education (Skaalvik and Skaalvik, 2009). Exploratory factor analysis of both item sets revealed a two-factor solution, with strong loadings (0.57–0.80) and moderate cross-loadings (0.38, 0.40) for two items, suggesting that the new scale captures a distinct sub facet of leader support. Inter-item correlations (*r* ≈ 0.30–0.55) further supported this distinction. The leader support scale itself showed strong loadings (>0.80) but poor initial model fit (*χ*^2^/df = 13.3, robust RMSEA = 0.256). However in the full models the scale worked well when residual covariances were correlated.

We also explored the associations to school contextual variables (organizational conditions) forming the nomological network, results of which are presented in Table 3.

**Table 3:** Nomological network.

Outcome	Dependent	χ^2^/df	CFI	TLI	RMSEA	SRMR	Construct	β (Std.)	*p*
Time Pressure (Model 1)	Collaboration Types	1.799	0.954	0.931	0.068	0.038	Exchange	0.472	0.003**
							Synchronization	−0.059	>0.5
							Co-Construction	−0.374	0.030*
Collegial Support (Model 2)	Collaboration Types	3.350	0.949	0.924	0.086	0.045	Exchange	0.699	<0.001**
							Synchronization	−0.485	<0.001**
							Co-Construction	0.331	0.008**
Shared Goals and Values (Model 2)	Collaboration Types	3.350	0.949	0.924	0.086	0.045	Exchange	−0.276	0.003**
							Synchronization	0.304	0.006**
							Co-Construction	−0.425	<0.001**
Time Pressure (Model 3)	Collaborative culture, leadership, contrived collegiality	2.663	0.956	0.937	0.067	0.048	Collaborative Leadership	0.158	0.002**
							Contrived collegiality	−0.092	0.102
							Collaborative Culture	−0.104	0.094
Collegial Support (Model 4)	Collaborative culture, leadership, contrived collegiality	2.460	0.953	0.933	0.080	0.037	Contrived collegiality	−0.343	<0.001**
							Collaborative Culture	0.252	<0.001**
							Collaborative Leadership	0.223	<0.001**
Shared Goals and Values (Model 4)	Collaborative culture, leadership, contrived collegiality	2.460	0.953	0.933	0.080	0.037	Contrived collegiality	0.178	0.001**
							Collaborative Culture	−0.146	0.003**
							Collaborative Leadership	−0.357	<0.001**

Robust indices for CFI, TLI and RMSEA are reported in this table.

### 3.4 Measurement invariance and reliability

For the invariance models across groups, we were unable to use school as a grouping variable because the clusters were too few and too small for reliable estimation. Instead, we used sex and age groups, with the latter defined as early-career (<35), mid-career (35–50), and late-career (>50) teachers.

For the collaboration types model using sex as the grouping variable, we found no significant worsening from the baseline (configural) to the weak factorial (metric) model based on the ANOVA test. The decrease in CFI was 0.001, and RMSEA showed a slight improvement in fit, supporting invariance across these models. The same pattern emerged when moving from the weak factorial to the strong factorial model, indicating that strong factorial invariance holds for this model when grouping by sex.

Using age groups, the ANOVA comparing the baseline and threshold model was non-significant, and both CFI and RMSEA remained within acceptable limits, indicating weak factorial invariance. The ANOVA for the strong factorial model was also non-significant, again supported by the fit criteria, leading to the conclusion that strong factorial invariance holds when using age as the grouping variable.

For the second set-ESEM model with the related collaboration constructs and sex as the grouping variable, we encountered convergence issues and therefore tested each construct separately. For all three constructs, ANOVA tests comparing the baseline and weak factorial models were non-significant, and CFI and RMSEA met invariance criteria. The same results were observed when comparing weak to strong factorial models, providing evidence of strong factorial invariance. These findings were replicated using age as the grouping variable, further supporting strong factorial measurement invariance across groups.

For longitudinal invariance testing, we modeled all factors separately due to the large number of parameters in threshold models and the associated risk of convergence issues. For exchange (collaboration types), the ANOVA comparing the baseline and weak factorial models was only non-significant at the 0.01 level (*p* = 0.01981), suggesting possible non-invariance. However, both CFI and RMSEA supported invariance, leading us to conclude that there is evidence for weak factorial invariance across waves. For the strong factorial model, none of the criteria were met, indicating no evidence of strong factorial invariance for this construct.

For Synchronization (collaboration types), all criteria supported strong factorial invariance. We found the same pattern for co-construction, leading to the conclusion that this construct also demonstrates strong factorial invariance across waves. However, this means that for the full multidimensional model including all three constructs, we may only claim evidence for weak factorial invariance across time points.

The related constructs model showed non-significant chi square difference test from baseline to weak factorial models. Delta CFI and delta RMSEA were also within established cutoffs. However, the strong factorial model failed to converge leading us to conclude that only weak factorial invariance holds for the model.

Reliability coefficients, inter-item and item-total correlations are summarized in Table 4.

**Table 4:** Reliability.

Construct/scale	Wave	Ω (Total)	Ωh (Hier.)	Zumbo’s Ordinal α	Inter-item r (range)	Item-total r (range)
Collaboration Types (Set-ESEM 1)	T1	0.79–0.81	0.79	0.78–0.82	0.23–0.77	0.53–0.82
	T2	0.77–0.83	0.74	0.75–0.83	0.26–0.79	0.51–0.82
Multidimensional scale (Collab)	T1	0.91	0.79	—	—	—
	T2	0.91	0.74	—	—	—
Related Constructs (Set-ESEM 2)	T1	0.85–0.95	0.55	0.85–0.94	−0.42–0.83	0.23–0.54
	T2	0.83–0.94	0.51	0.83–0.93	−0.39–0.82	0.11–0.54
Multidimensional scale (Related)	T1	0.93	0.55	—	—	—
	T2	0.92	0.51	—	—	—

## 4 Discussion

In relation to the first research question, the initial correlation plots revealed clear and theoretically coherent clustering, with items grouping according to the expected factor structures. These patterns provide early support for discriminant validity, as they align with the theorized measurement models. With only a few exceptions, the ESEM models demonstrated good fit, offering evidence of convergent validity. We retained six factors that represent constructs that were both empirically stable and theoretically distinct. Each factor demonstrated acceptable convergence, minimal cross-loadings, and a clear interpretive structure, in contrast to other candidate constructs that exhibited instability or conceptual overlap. Importantly, the retained factors reflect the two complementary theoretical foundations guiding this study. The first set aligns with the typology of collaborative acts by Gräsel et al. (2006), capturing the behavioral dimensions of teacher collaboration, while the second set builds on Hargreaves’ work (2019; 2018), encompassing cultural and organizational aspects that support collaborative practice (collaborative leadership, collaborative culture, contrived collegiality). Together, these six factors preserve the breadth of the conceptual framework while maintaining psychometrically robust measurement models.

Regarding content validity, the items reflected their intended conceptual domains: Set 1 captured the narrow typology of collaboration practices, while set 2 represented broader school-level conditions relevant to collaborative processes. Consistent with Gräsel et al.’s (2006) theoretical framework of collaboration typology, Set 1 items aligned well with the corresponding three-dimensional factor structure.

Within the ESEM framework, lower standardized primary loadings are generally expected compared to traditional CFA models due to the allowance of cross-loadings. Accordingly, loadings ≥ 0.30 were considered acceptable, provided those primary loadings remained clearly distinguishable from cross-loadings. As anticipated, comparison with CFA models showed stronger factor loadings (all exceeding 0.50), yet the ESEM loadings were still sufficient to support the factor structure. The three facets were meaningfully interrelated, with factor covariances around 0.6.

Latent factor correlations in the ESEM models (0.65–0.69) were lower than those in the corresponding CFA models (0.77–0.86), reflecting the redistribution of shared variance through cross-loadings and providing a more realistic representation of factor overlap. This level of association indicates shared variance consistent with convergent validity while remaining moderate enough to demonstrate discriminant validity. This interpretation aligns with previous recommendations and findings within the ESEM framework (Alamer, 2022; Alamer and Marsh, 2022; Morin et al., 2020).

For the broader collaboration-related constructs in set 2, intercorrelations were smaller, suggesting weaker justification for a unified general dimension and greater discrimination among these constructs compared with set 1. Nevertheless, the simple structure was clearer in set 2, with stronger factor loadings and lower latent factor correlations, indicating clearer convergence and discriminant validity.

Regarding the second research question, the latent constructs demonstrated meaningful and theoretically relevant associations with burnout and engagement supporting criterion validity. Collaboration types showed clear relationships with burnout and, to a lesser extent, engagement. Among them, exchange exhibited the strongest pattern, with small to moderate negative associations with all dimensions of burnout except cognitive impairment, and moderate positive associations with engagement. This pattern may reflect that a collectively oriented work environment, characterized by frequent professional exchanges, has protective effects – consistent with previous findings on the importance of peer support (Skaalvik and Skaalvik, 2007; Skaalvik and Skaalvik, 2021).

Synchronization showed a small positive association with burnout, potentially reflecting the coordination demands inherent in collaborative work; controlling for time pressure did not alter this pattern. Emotional exhaustion (MBI) demonstrated similar, albeit slightly weaker, associations with collaboration types. Engagement (UWES) showed weak but significant positive associations with synchronization.

Contrived collegiality consistently displayed positive relationships with aspects of burnout and negative relationships with work engagement, supporting theoretical assumptions that contrived collegiality may adversely affect teachers’ well-being and contribute to disengagement (Hargreaves, 2019; Hargreaves and O’Connor, 2018). Collaborative leadership showed consistently negative associations with burnout, reflecting the well-documented importance of leader support for teacher well-being (Ford et al., 2019).

Although collaborative culture was not significantly associated with the BAT, it was significantly negatively associated with emotional exhaustion (MBI) and positively associated with work engagement. Overall, collaboration types, collaborative leadership, collaborative culture, and contrived collegiality exhibited consistent associations with burnout and engagement, providing robust evidence for criterion validity.

Concurrent validity was examined by incorporating auxiliary variables into the two ESEM models. Some findings diverged from theoretical expectations – for example, time pressure showed a moderate positive association with exchange, despite assumptions of a negative relationship. This may indicate that more frequent transactions reduce available time for core job tasks. In contrast, co-construction exhibited a small to moderate negative correlation with time pressure, confirming that deeper, more complex collaborative activities can reduce teacher workloads, as reported in previous studies (Muckenthaler et al., 2020).

For the broader constructs in set 2, associations with time pressure were generally smaller and mostly insignificant, except for a significant positive association with collaborative leadership. This suggests that more direct leader involvement in collaboration may increase teachers’ time pressure.

Collaboration types showed moderate to strong associations with collegial support in both directions. Specifically, collegial support was strongly positively associated with exchange, moderately negatively associated with synchronization, and showed a small to moderate positive association with co-construction. This pattern suggests that low-cost, frequent exchanges may be particularly important in fostering positive relationships between teachers, which has been linked to job satisfaction (Skaalvik and Skaalvik, 2011). Collegial support also demonstrated consistent associations with the constructs in the second set of ESEM models.

Contrived collegiality displayed a moderate negative association with collegial support, providing further evidence for Hargreaves’ theory regarding the potential negative effects of inauthentic collaboration (Hargreaves and O’Connor, 2018). Shared goals and values, however, showed a small negative association with collaborative culture, indicating potential divergence between goal orientation at the team level versus the school-wide level. Although both variables relate primarily to goal orientation, collaborative culture is more closely tied to team level goals, which may also explain the moderate negative association between shared goals and values and collaborative leadership which also is related to team level activity.

Additional evidence of discriminant validity for collaborative leadership emerged from its differential associations with supervisory support and shared goals and values. While supervisory support and collaborative leadership shared some variance and cross-loadings, the distinction observed suggests that collaborative leadership is not redundant. Instead, it represents an additional leadership facet specifically related to collaborations. This construct may also relate to other factors previously associated with supervisory support, including collective teacher efficacy, job satisfaction, and instructional quality (Skaalvik and Skaalvik, 2021).

In sum, nomological and concurrent validity was supported by these generally consistent relationships between collaboration types, contrived collegiality, collaborative culture, and collaborative leadership with the external variables included in the study.

Turning to the third research question, the invariance analyses provide further support for the validity of the collaboration measures. Strong factorial invariance across teacher subgroups, both by sex and age, suggests that the constructs are interpreted consistently across different demographic segments of the teaching workforce. This strengthens confidence that the scale captures generalizable features of collaborative practices rather than subgroup-specific interpretations. Similar findings have recently been reported for a related scale introduced earlier, where the expanded co-construction scale demonstrated invariance across demographic groups (Kluge et al., 2025). This provides evidence that the concepts underlying this typology of teacher collaboration may also be invariant across cultural contexts.

The longitudinal tests revealed a more differentiated pattern. Synchronization and co-construction demonstrated strong factorial invariance across measurement occasions, indicating stability in how teachers understand these forms of collaboration over time. Exchange, however, showed only weak invariance. This suggests that although the construct remains identifiable across waves, the thresholds for item responses may shift. Such shifts may reflect changes in contextual demands, seasonal fluctuations in collaborative routines, or evolving interpretations as collaborative practices unfold across the school year.

Taken together, the findings provide a coherent and multi-layered validation of the proposed measurement model of teacher collaboration. The factor structure was largely stable and aligned with theoretical expectations, the constructs showed meaningful and theoretically relevant associations with external variables and outcomes, and the measures demonstrated strong invariance across teacher subgroups. Although longitudinal tests revealed variability, particularly for exchange, the overall pattern indicates that the core dimensions are interpreted consistently within and across groups, with some expected sensitivity to temporal and contextual changes. These results suggest that the measurement model captures substantively important and generalizable aspects of collaborative practice in Norwegian primary education, offering a solid foundation for future research and applied work aimed at understanding how collaboration relates to key indicators of teacher well-being.

Reliability analyses across both waves indicated that the individual constructs in the collaboration types model were highly consistent, with Omega and ordinal alpha values above 0.77. For the multidimensional scale, total common variance reliability was high (0.91), and hierarchical Omega was also substantial (0.74–0.79), suggesting that a general factor accounts for a meaningful proportion of variance in this scale. For the related constructs model, subscales were similarly reliable (Omega and ordinal alpha ≥0.83), but hierarchical Omega for the overall multidimensional scale was low (0.51–0.55), indicating that the general factor explains only a modest proportion of variance. In both models, inter-item and item-total correlations supported good coherence within subscales. These findings suggest that while subscales are highly reliable, the overall total scores are more meaningful for collaboration types than for the related constructs, consistent with the multidimensional nature of the measures.

## 5 Implications, limitations and future studies

### 5.1 Implications

A key advantage of the measures developed in this study is their brevity. They make it possible to study different types of collaboration without placing heavy demands on survey space. Although longer existing instruments may capture collaboration in greater detail, they are often impractical for large-scale studies. We therefore recommend our measures as efficient tools for assessing teachers’ collaboration in large surveys.

One of the main implications of this study is the variation in relations between different types of collaboration and related constructs on the on hand, and external variables on the other. This shows that sub facets of teacher collaboration differ significantly with regards to other constructs. This has some important implications with regards to leadership and organizational strategies and may nuance popular impressions that collaboration is a simple construct that is either positive or negative.

The invariance findings are particularly noteworthy given the lack of established collaboration scales in the Nordic context. Demonstrating stable measurement across teacher groups and (for two constructs) across time suggests that the adaptation of the German typology is psychometrically appropriate for Norwegian schools and potentially transferable to similar educational systems.

The partial longitudinal invariance has implications for interpreting the associations between collaboration and outcomes. With weak invariance for exchange, comparisons of latent means across time should be made cautiously, whereas associations with external variables remain interpretable. For synchronization and co-construction, the evidence of strong invariance supports more confidence in both longitudinal comparisons and structural relations.

### 5.2 Limitations and future studies

Caution is warranted when interpreting results from invariance models involving collapsed categories, as this approach may partially violate underlying statistical assumptions. Collapsing categories introduces several possible limitations. First, it reduces variance, which can weaken correlations among items, lower factor loadings, and decrease the precision of parameter estimates. Second, distributional properties may be affected, although robust estimation mitigates this to some extent. Third, fewer response categories can make it harder to detect subtle group differences, limiting the sensitivity of cross-sectional multigroup invariance tests. We note that longitudinal invariance models were conducted without collapsing categories and yielded satisfactory results. Nevertheless, we recommend caution when interpreting the cross-sectional multigroup invariance findings, due to these limitations.

One important limitation of the present study is the lack of norming for the developed scales. Future research using diverse samples is needed to confirm the factorial structure and establish normative benchmarks. Moreover, the weaker longitudinal performance of the exchange construct highlights the need for further refinement, especially for items capturing routine, low-intensity collaboration. Such practices may be more sensitive to contextual fluctuations, suggesting that future work should explore whether additional or alternative indicators might capture the construct with greater temporal stability.

Multivariate latent normality was not supported, as indicated by results from the discnorm() package. However, the extent of non-normality remains unknown, as no established method currently exists to quantify this in ordinal data. Consequently, some bias may be present due to the violation of latent normality assumptions. Given the importance of this issue for researchers working with ordinal data, further methodological development is needed to assess the degree of underlying non-normality.

Another limitation concerns the directionality of relationships between collaboration constructs and burnout. While cross-sectional associations were observed, longitudinal research is needed to clarify how teacher collaboration is related to burnout and engagement over time. Future studies should explore these dynamics to better understand the temporal and possible causal nature of these relationships.

Some constructs, particularly those related to collaborative leadership, exhibited moderate intraclass correlations (ICCs = 0.12–0.17), indicating non-negligible clustering of responses within schools. Due to the small number of clusters and limited respondents per school, it was not feasible to model these constructs using multilevel approaches. Consequently, the standard errors for these items may be somewhat underestimated, and results should be interpreted with caution, particularly for between-school comparisons. Future studies with larger and more evenly distributed samples should consider multilevel modeling to account for school-level clustering.

ESEM allows simultaneous exploration and confirmation of the factor structure, mitigating some limitations of traditional CFA. However, because the same sample is used for both model development and estimation, overfitting remains a potential concern and replication in an independent sample is therefore recommended.

## Data Availability

The raw data supporting the conclusions of this article will be made available by the authors, without undue reservation.

## Footnotes

1 Note that we only used dataframes with collapsed categories (from 5 to 4) for the invariance models across groups and for one of the longitudinal ESEMs. All other models, including longitudinal invariance models, were done with data that did not have collapsed categories.

2 Software versions: R (v4.4.1), psych (v2.4.6.26) and lavaan (0.6–19).

3 Longitudinal ESEM with ordinal estimation entails a very high number of parameters per item in the model. This can make estimation challenging and often leads to non-convergence issues. Switching estimator will often lead to some loss of precision regarding SEs and parameter estimates.
